# Supplementary figures and images for: One dog’s waste is another dog’s wealth: A pilot study of fecal microbiota transplantation in dogs with acute hemorrhagic diarrhea syndrome
Source: PLoS One. 2021 Apr 19;16(4):e0250344. doi: 10.1371/journal.pone.0250344 (PMC8055013; doi:10.1371/journal.pone.0250344)

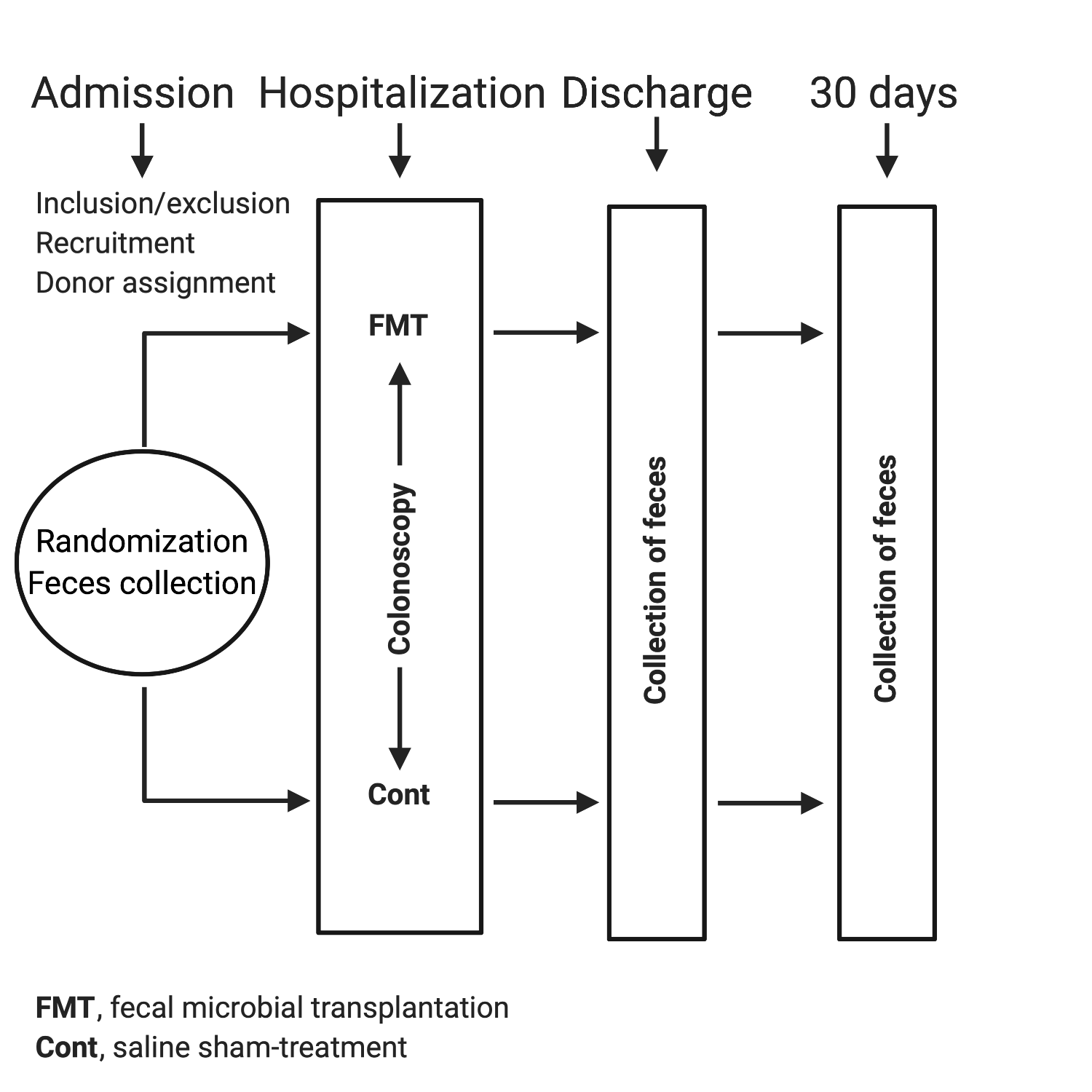

Supplement: S1 Fig — Following inclusion of dogs with AHDS to the study and collection of their feces, the dogs were randomized to undergo a colonoscopy procedure during which they received either FMT or sham treatment with saline. The following day, the dogs with AHDS had a colonoscopy procedure. A portion from each of the donors’ feces used for FMT was saved. Feces were re-collected from the donors, FMT-recipient dogs with AHDS and sham-treated dogs with AHDS at the time of discharge and 30 days after discharge. AHDS: acute hemorrhagic diarrhea syndrome; FMT: fecal microbial transplantation. (TIF) [file pone.0250344.s001.tif]

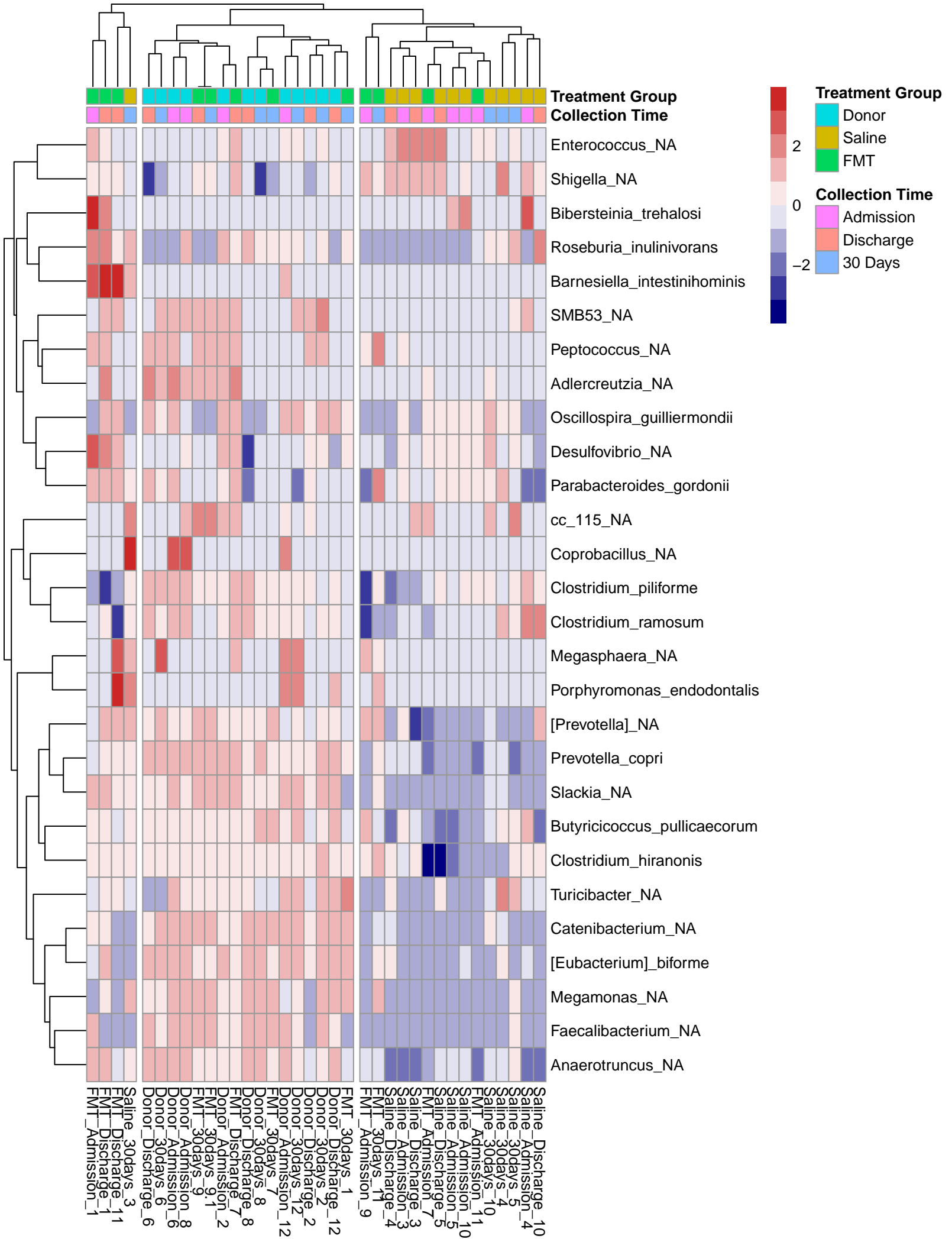

Supplement: S2 Fig — AHDS: acute hemorrhagic diarrhea syndrome; FMT: fecal microbial transplantation. (PDF) [file pone.0250344.s002.pdf]
