# Supplementary material for: One dog’s waste is another dog’s wealth: A pilot study of fecal microbiota transplantation in dogs with acute hemorrhagic diarrhea syndrome
Source: PLoS One. 2021 Apr 19;16(4):e0250344. doi: 10.1371/journal.pone.0250344 (PMC8055013; doi:10.1371/journal.pone.0250344)
Supplement: S1 Table — (DOCX) [file pone.0250344.s003.docx]

| **S1 Table**. Criteria for clinical assessment of acute hemorrhagic diarrhea syndrome clinical score | | | | | |
| --- | --- | --- | --- | --- | --- |
| Score | Activity | Appetite | Vomiting^*^ | Fecal consistency | Defecation^*^ |
| 0 | Normal | Normal | 0 | Normal | 1 |
| 1 | Mildly reduced | Mildly reduced | 1 | Slightly soft | 2-3 |
| 2 | Moderately reduced | Moderately reduced | 2-3 | Very soft | 4-5 |
| 3 | Severely reduced | Severely reduced | >3 | Watery | >5 |
| Relationship between total score and clinical significance: **0-3**, insignificant; **4-5**, mild; **6-8**, moderate; **> 8**, severe    ^*^Per day    Adopted from Mortier F et al. [1]. | | | | | |

Reference

1. Mortier F, Strohmeyer K, Hartmann K, Unterer S. Acute haemorrhagic diarrhoea syndrome in dogs: 108 cases. Vet Rec. 2015;176(24):627.
