# Supplementary material for: One dog’s waste is another dog’s wealth: A pilot study of fecal microbiota transplantation in dogs with acute hemorrhagic diarrhea syndrome
Source: PLoS One. 2021 Apr 19;16(4):e0250344. doi: 10.1371/journal.pone.0250344 (PMC8055013; doi:10.1371/journal.pone.0250344)
Supplement: S2 Table — (DOCX) [file pone.0250344.s004.docx]

| **S2 Table:** Primer sequences for 16S-rRNA amplicon sequencing | |
| --- | --- |
| **Primer** | **Sequence** |
| 16Sf V3 | AATGATACGGCGACCACCGAGATCTACACxxxxxxxxTATGGTAATTGGCCTACGGGAGGCAGCAG |
| 16Sr V4 | CAAGCAGAAGACGGCATACGAGATxxxxxxxxAGTCAGTCAGCCGGACTACHVGGGTWTCTAAT |
