# Supplementary material for: One dog’s waste is another dog’s wealth: A pilot study of fecal microbiota transplantation in dogs with acute hemorrhagic diarrhea syndrome
Source: PLoS One. 2021 Apr 19;16(4):e0250344. doi: 10.1371/journal.pone.0250344 (PMC8055013; doi:10.1371/journal.pone.0250344)
Supplement: Attachment — Submitted filename: Response to Reviewers.docx [file pone.0250344.s006.docx]

The authors thank the editor and both Reviewers for the time they invested in reviewing the manuscript and providing their valuable comments and suggestions for improving the manuscript and making it more impactful. Below we provide a point-by-point responses to each of the comments made by the Reviewers and editor.

EDITOR’S COMMENTS:

1. Please ensure that your manuscript meets PLOS ONE's style requirements, including those for file naming. The PLOS ONE style templates can be found at

<https://journals.plos.org/plosone/s/file?id=wjVg/PLOSOne_formatting_sample_main_body.pdf> and

<https://journals.plos.org/plosone/s/file?id=ba62/PLOSOne_formatting_sample_title_authors_affiliations.pdf>

Authors response: In the resubmission we believe that we complied with PLOS ONES’s style requirements. The file names were changed according to <https://journals.plos.org/plosone/s/file?id=wjVg/PLOSOne_formatting_sample_main_body.pdf>

We apologize for not paying attention to file naming aspect in the original submission.

2. Please confirm that the authors obtained consent from owners to have their dogs participate in the research reported in this paper.

Authors response: We confirm that the owners of the patients enrolled in the study gave a written consent to enroll their pets in the study.

3. Thank you for stating the following in the Financial Disclosure section:

'This study was funded by the Massey University’s College of Science Research Fund (MURF). The funders had no role in study design, data collection and analysis, decision to publish, or preparation of the manuscript.' We note that one or more of the authors are employed by a commercial company: Small Animal Medicine Specialist Services, Townsville.

a. Please provide an amended Funding Statement declaring this commercial affiliation, as well as a statement regarding the Role of Funders in your study. If the funding organization did not play a role in the study design, data collection and analysis, decision to publish, or preparation of the manuscript and only provided financial support in the form of authors' salaries and/or research materials, please review your statements relating to the author contributions, and ensure you have specifically and accurately indicated the role(s) that these authors had in your study. You can update author roles in the Author Contributions section of the online submission form.

Please also include the following statement within your amended Funding Statement.

“The funder provided support in the form of salaries for authors [insert relevant initials] but did not have any additional role in the study design, data collection and analysis, decision to publish, or preparation of the manuscript. The specific roles of these authors are articulated in the ‘author contributions’ section.”

If your commercial affiliation did play a role in your study, please state and explain this role within your updated Funding Statement.

Authors response: Dr. Burchell was employed at Massey University during the time of the study. He left after the study was completed and has been working in a private referral center. His current employment had no impact on the study whatsoever. Like many private practitioners involved in research his involvement in the paper subsequent to leaving the university sector has been in his own time.

b. Please also provide an updated Competing Interests Statement declaring this commercial affiliation along with any other relevant declarations relating to employment, consultancy, patents, products in development, or marketed products, etc.

Within your Competing Interests Statement, please confirm that this commercial affiliation does not alter your adherence to all PLOS ONE policies on sharing data and materials by including the following statement: "This does not alter our adherence to  PLOS ONE policies on sharing data and materials.” (as detailed online in our guide for authors [http://journals.plos.org/plosone/s/competing-interests](about:blank)) . If this adherence statement is not accurate and there are restrictions on sharing of data and/or materials, please state these. Please note that we cannot proceed with consideration of your article until this information has been declared.

Authors response: The authors declare no conflict of interests. Dr. Burchell’s current affiliation did not conflict with preparation of the manuscript.

c. Please include both an updated Funding Statement and Competing Interests Statement in your cover letter. We will change the online submission form on your behalf.

Authors response: We provided the following in the cover letter: “Also as requested by the Associate Editor, we amended our Funding Statement. We declare that the funder (i.e., Massey University) provided support in the form of salaries for authors [AG, RB, PB, KG and AM], but did not have any additional role in the study design, data collection and analysis, decision to publish, or preparation of the manuscript. The specific roles of these authors are articulated in the ‘author contributions’ section. Dr. Burchell’s current affiliation did not alter our adherence to  PLOS ONE policies on sharing data and materials”.

Please know it is PLOS ONE policy for corresponding authors to declare, on behalf of all authors, all potential competing interests for the purposes of transparency. PLOS defines a competing interest as anything that interferes with, or could reasonably be perceived as interfering with, the full and objective presentation, peer review, editorial decision-making, or publication of research or non-research articles submitted to one of the journals. Competing interests can be financial or non-financial, professional, or personal. Competing interests can arise in relationship to an organization or another person. Please follow this link to our website for more details on competing interests: [http://journals.plos.org/plosone/s/competing-interests](about:blank)

Authors response: During the time that the study was conducted Dr. Richard Burchell was a faculty at Massey University, and did not have any conflict of interests.

We did not find anywhere in PLOS ONE Editorial system during resubmission a place where we could amend the Competing Interests Statement. In the ‘manuscript data tab’ under ‘Funding information’ there was no area to amend what the editor requested. The roles of each contributor accurately reflect their involvement at different aspects that were listed.

**REVIEWERS COMMENTS:**

**FIRST REVIEWER COMMENTS:**

This study describes the clinical and faecal bacteriome characteristics of a series of dogs that presented with acute haemorrhagic diarrhea and were treated with a faecal microbial transplant or saline placebo. The understanding of bacterial disturbances associated with, and causing disease (dysbiosis) in dogs is foetal at best, and barely embryonic in regards to the response to FMT. This study, though very small, describes changes to the bacteriome in clinical cases, and represents an important contribution to a very empty landscape. The study was well designed (with one significant criticism), is methodologically strong, well presented, and whilst the analysis bordered on excessive given then small number of dogs, it was conducted to a high standard. It is very unlikely that we will develop a general understanding of the contribution of the bacteriome to health and disease for some time to come, and it will require the assimilation of a large number of studies that include a wide variety of dogs, diets, and clinical diseases, and as such, this present study will undoubtably become part of that canon.
Authors response: Thank you for seeing the significance of this study. We agree with the Reviewer that there is an overall need to increase the number of well-designed studies on companion animal microbiome in general and on AHDS in particular. We think that our study provides important information that generated new knowledge. We agree with the Reviewer that multiple contributing factors participate in the evolution and resolution of AHDS and we added relevant comments (following the Reviewers’ suggestions below) throughout the manuscript. We also thank the Reviewer for seeing beyond the limitation of a small sample size and for not automatically discounting the important data in the present study.

**Major criticisms**
I have two major criticisms that I would appreciate the authors address. One is conceptual and relates more to the discussion and interpretation, and the other is a methodological limitation. Firstly, the authors refer to, and base their analysis on the concept that AHDS is a syndrome with features that are common to most, if not all cases. The manuscript starts, not with a description of AHDS or a defense of it as a unitary disease, but with a description of recurrent C.difficile enterocolitis in humans. It may warrant comparison with the disease in humans at some point in the discussion, but in this Reviewer’s opinion they are not sufficiently similar as to justify comparison in the introduction. That would be a small point were it not for the fact that it sets the tone for manuscript and the interpretation of the data. The dogs included in this study, and indeed in others that have preceded it, do not have recurrent disease, nor do they have overgrowth of a clostridial species, let alone difficile. In fact, on face value, this study more strongly suggests that AHDS is not caused by a dysbiosis than that it is. I would want the authors to consider these dogs as sharing some common clinical features, but certainly not necessarily a common aetiology, and thus one would not expect there to be a common cure. Whilst I do not claim that a microbial disturbance is not causal in AHDS, I claim that a) there are many potential causes of the syndrome, b) the abnormalities described in this paper may well be secondary and not causal, and c) the findings of this paper provide evidence against a microbial hypothesis, are discordant with previous studies, and certainly do not support any similarity with C.diff infections in humans.

Secondly, the intestinal microflora is the product of bacteria that are introduced into it, and the substrate that is provided, and modified by host and community factors. The authors have only considered the bacteria introduced through the FMT, and have not reported, or apparently considered the effects of substrate. Numerous studies have shown the profound effect of diet on the bacteriome, and it is very likely that any sustained change effected by FMT in a given patient will be a complex interaction between host, disease, host microflora, donor microflora, and diet – both preceding and following transplantation. I am sure that other readers will be surprised that no effort was made to either control, or even describe the post-treatment diet, and it is a major oversight not to consider its effects.
Authors response: We acknowledge that every study has limitations, especially clinical studies such as the present study. We did our best to adapt the suggestions made by the Reviewer and to highlight and address our study’s limitations where necessary. Below we provide specific responses to the Reviewer’s major critique.

RESPONSE TO THE FIRST LIMITATION HIGHLIGHTED BY THE REVIEWER: The authors regard AHDS as a clinical syndrome, hence the authors have a similar view to that of the Reviewer that AHDS has set of clinical signs and clinicopathologic abnormalities which are correlated with each other and associated with a particular clinical presentation. The etiology of AHDS is unclear and different studies pointed out involvement of different bacteria, bacterial toxins, and dietary components which had been found in association with AHDS ^1-9^. Since Koch’s postulates have not been fulfilled with regards to AHDS, a definitive etiological cause is unknown at this point. We suspect that there isn’t a single causative etiology for AHDS but rather a constellation of different host, microbiome, and environmental conditions that, in a given undefined moment lead to onset of clinical disease. We rewrote the first two paragraphs of the introduction to reflect this view.

Our intention in comparing AHDS to *C. Difficile* infections in humans early in the introduction was to set the tone towards the profound clinical effect that fecal microbial transplantation might have in a disease process (complex and multifactorial as it might be) where the histopathology pattern of intestinal mucosal injury resembles the one in AHDS and thus could suggest a central role for a toxin-mediated intestinal mucosal injury. Our notion is supported by the literature on AHDS ^4,5,7,8^ and the limited studies from which intestinal histopathology from patients with AHDS has been published ^5,7,10^. The histopathologic pattern is similar to those of other diseases in which there is bacterial toxin-mediated mucosal necrosis ^11,12^. Antibiotic-mediated **dysbiosis** is thought to be a critical factor in the induction of *C. Difficile* proliferation and toxin release. While the Reviewer highlighted the differences between AHDS and *C. Difficile* infections (about which we agree with the Reviewer) the fact that FMT is so effective in *C. Difficile* infections is why we chose to contrast it with AHDS in the pre-revised manuscript version. That being said, we completely understand the Reviewer point-of-view and in accordance with the Reviewer, we made substantial accommodating changes in the introduction and discussion sections.

We agree with the Reviewer that we cannot claim that AHDS is caused by a dysbiosis. Our study was not designed to assess causation. However, its longitudinal nature allowed us to follow the AHDS FMT recipient and control dogs beyond the resolution of clinical disease and we documented persistent alterations in microbial community composition in these dogs (when compared to the healthy donors). Alpha and beta diversity are accepted global indices used in the evaluation of a state of gut dysbiosis. The fact that dysbiosis persisted in the control dogs, and after 30 days, also in treated dogs, could suggests that other factors may predispose to gut dysbiosis (again in agreement with the Reviewer’s views) but that does not mean that gut dysbiosis might not be central in the development of the clinical disease.

RESPONSE TO THE SECOND LIMITATION HIGHLIGHTED BY THE REVIEWER: The authors admit upfront that not considering controlling the diet is indeed a limitation. If we could redesign the study, we would have paid a lot more attention to the dietary aspect. Yet, the Reviewer might also consider that in a clinical trial that is intended to simulate real-life scenario of FMT treatment in AHDS dogs, leaving the diets uncontrolled would better reflect what the efficacy might be in the real world. Clinical FMT in people is also done without controlling the diets (in the human FMT trials that we reviewed the diet was not controlled). We also think that the Reviewer would agree that in the setting of the peracute onset of clinical presentation in AHDS it is also impossible to acclimate dogs to a unified diet. The authors cannot agree more with the Reviewer’s statement that “any sustained change effected by FMT in a given patient will be a complex interaction between host, disease, host microflora, donor microflora, and diet – both preceding and following transplantation”. Lastly, given the limitation that we had i.e., a clinical study on a disease with a low incidence that had to be completed within a year to adhere to the funding guidelines of the funding body, we could not be selective with regards to the pre-enrollment nutrition status of the patients. We discussed this issue in lines 548-556.

Specific criticisms
Line 50: It is no clear what the relevance of the comparison with RCI is, especially in light of the author’s own data which disprove any similarity.

Authors response: the introduction was restructured and RCI is just mentioned in the context of a disease in people in which there is strong evidence for efficacy of FMT.

Line 62: I disagree with general statements regarding the “dysbiosis of AHDS”. Two studies have shown some differences, though the two cited studies unsurprisingly differed. In keeping with the lack of conformity, none of the taxa that were most different in the study by Suchodolski et al, were altered in the author’s study. Thus, their own data prove that there is no a generalizable feature of the bacteriome in this syndrome. In fact, it remains to be seen if the clinical presentation of these dogs has a common aetiology, and if it does, it does not appear to be due to consistent changes in the bacteriome.

Authors response: In a broad sense, changes in alpha and beta diversity between healthy control population and diseased patients represent gut dysbiosis. In our opinion there isn’t a single causative etiology or specific pathobiont for AHDS but rather a constellation of host, microbiome, and environmental conditions unique to the individual dog that in a given undefined moment ripen and lead to the onset of clinical disease.

In Dr. Suchodolski’s study the AHDS dogs had a significant decreased differential abundances of the SCFA-producing taxa Erysipelotrichaceae (i.e., genus Turicibacter), Ruminococcaceae (i.e., Ruminocococcus, Faecalibacterium) and Lachnospiraceae (i.e., Blautia). In our study there was also a significant decreased differential abundance of important SCFA-producing taxa (Table 3). We do not suggest that dysbiosis in AHDS is homogenous. We do not think that the deviation from normal (i.e., normobiosis) should not be considered abnormal (i.e., dysbiosis) just because the changes in communities composition in our study were not of the exact same taxa as those described in Dr. Suchodolski’s study or in the other study that we referenced.

We emphasize that we found an association between a condition (i.e., AHDS) and changes in alpha and beta diversity. We are not pointing out a cause-and-effect relationship. To make it clear we added the following sentences In lines 53-57 “Evidence suggests that dogs with AHDS might also have gut dysbiosis [4, 10, 12], however, evidence differs with respect to the composition of gut bacterial communities, thus highlighting the inherent heterogeneity of this syndrome. To date, a direct cause-and-effect relationship between AHDS and specific bacterium, a state of gut dysbiosis, or bacterial toxins has not been established.”

However, at the same time we caution against categorically stating that decreased differential abundance of SCFA-producing taxa (as a group and not specific taxa) in both studies and ours might not represent a potentially significant risk factor for development of AHDS. Nonetheless, we think that the more data we generate on AHDS dogs from diverse backgrounds (e.g., breed, age, sex, neuter status, countries, diets, pre-existing medical conditions, physical environments) the more we are likely to stumble on generalizable common denominators that are critical for the development of this clinical syndrome.

Line 73: Why would the authors not have hypothesized that FMT would have a clinical benefit? I find it hard to believe that this study was conducted with the intention simply to look at the faecal bacteria, and it has the sense of post hoc hypothesis creation, since the treatment had no clinical benefit. I think to avoid suspicion of such post hoccery, the authors need to either explain why a clinical benefit was of no interest, or include that hypothesis, which after all, the authors do go on to test anyway. If no clinical benefit was expected, what is the justification of the study other? And given that most dogs with idiopathic AHDS recover rapidly anyway with supportive care, what sort of clinical benefit were the authors trying to detect? What case numbers would be necessary to be able to detect a small difference in time to improvement?

Authors response:

For the purpose of power / sample size calculation, detecting a significant difference between two means of the variable ‘day of discharge from the hospital*’* assuming Δ(µ1-µ2) = 2d and σ = 1d, with an alpha probability error of 0.05 and a power of 0.8, can be achieved with 4 dogs in each group (**a** **total of 8 dogs**; calculated by G*Power software version 3.1.9.2). Based on the general Massey veterinary teaching hospital small animal population the year preceding the study (4095 cases) and the reported incidence of disease (0.51%-0.87%^1,13^), we expected to see ~35 cases of canine AHDS per 12 months of the study.

Unfortunately, we only enrolled one dog in the first 6 months of the study, and this is when we reached out to our colleagues in South Africa that have a higher incidence of AHDS. Thus, we managed to enroll a total of 4 dogs in each group. In the revised manuscript we added the hypothesis part and a description of the a priori power analysis that we did (lines 234-236). We also included a section in the discussion explaining that the study was under powered to detect a clinical benefit below a difference of two days in discharge (lines 542-545).

Line 82 and 85: Please change these written numbers “Seven and 1”. It sounds like something from the old testament!

Authors response: We will refer to the editor’s decision as we think that we followed the journal format.

Table 1. It is interesting that one of the donor dog’s was consuming Hill’s metabolic. Was the dog obese? That is a very high fibre diet compared with the other diets, and is known to modify the bacteriome compared with a standard diet. Why was the French Bulldog being fed z/d? Did it have a prior history of diarrhea? It is disappointing that there was no dietary history for most of the recipients. The authors cannot say if differences at the time of admission are not due to dietary differences and be unrelated to disease. Whilst I do not suspect that, it cannot be excluded, and should be discussed.

Authors response: We agree that this is a limitation and we discuss it under study limitations (lines 548-556): “Secondly, the authors acknowledge that not controlling for the diets of the dogs included in the study (both donors and AHDS dogs) is a limitation of the study, as the effect of FMT in a given patient is likely a complex interaction between the host genetic makeup and immune system, host microbiome, donor microbiome, and diet – both preceding and following FMT. However, we argue that if FMT were to be widely used in a clinical setting, controlling the diet would not be possible for practical reasons. We also argue that while the diets differed between dogs, the AHDS dogs clustered together before FMT and significantly diverged in accordance to FMT at the time of discharge (Fig. 4). Since everything other than FMT was held constant, we argue that the significant change in community composition was mostly contributed by FMT.”

We did not have any control over decisions that vets and owners made regarding the nutrition of the dogs in this study. Specifically, the dog on z/d diet had a history of skin allergies and this is why its primary care veterinarian decided to put it on z/d diet (i.e., suspected food allergy). Over conditioning of one of the donor dogs (BCS 6/9) was the reason for putting it on the Hill’s metabolic diet.

Line 93: Were faeces graded? How severe was the diarrhea?

Authors response: Consistent with a previous study from which we adapted our scoring system^14^, we scored feces from 0-3. On admission, all AHDS dogs had watery bloody diarrhea.

Line 94: Please better describe the disease definition. How was “haemorrhagic diarrhoea” diagnosed? What constituted “haemoconcentration”?
What other differential diagnoses were considered and excluded? Addisons? Toxicity? Pancreatitis? How was endoparasitism excluded?
Authors response: Hemorrhagic diarrhea was defined as large volume of a watery bloody diarrhea. Hemoconcentration was defined as packed-cell volume or hematocrit at or above the high end of the reference interval. All dogs had >60% except one with 55% which is the high end of the reference range at the University of Pretoria Veterinary Academic Hospital. In each of the institutions, cases were evaluated by a board-certified internist (AG, PP) who reviewed the clinical history, performed a physical exam, and interpreted the CBC/chemistry/urinalysis, and abdominal imaging. Endoparasites were excluded based on a fecal float. The signalment, history, clinical presentation, response to treatment and subsequent 1 month follow-up did not fit with Addison’s, toxicity or pancreatitis and we did not recruit any patient if we had a suspicion that it might have any conditions other than AHDS.

Line 104: The authors are probably aware of the rapid shifts in viable bacteria, especially anaerobic species, following defaecation, a process that is greatly accelerated with delays in handling, and inadvertent aeration of the samples. The reader requires more detail in the process of faecal handling. What quantity of faeces was collected? Were they placed immediately into RNAlater? Also, there is stratification of species from mucosa to lumen. When swabs or loops are used to collect samples, there is a bias towards mucosally-adherent species, when compared with the faecal bacteriome. Thus, when collected directly from the rectum, the nature of the specimen is influenced by the rectal contents. How was that considered/safeguarded against?

Authors response: Immediately after collection of feces with a fecal loop, 1 mL of feces were mixed inside a cryovial with 1 mL of RNAlater. We acknowledge that introducing a fecal loop that would potentially be in contact with the mucosa could bias towards mucosa-associated bacteria. This bias however is not reflected in our results.

Line 105-106: Please clarify if samples from dogs with AHDS were collected in a manner that differed from donor dogs or not.

Authors response: A portion (1mL) of the stool of the donors was mixed inside a cryovial with 1 mL of RNAlater immediately after defecation. Donors feces was not collected via a loop but following natural defecation.

Line 112: AHDS is, as the authors note, a syndromic diagnosis, and not one that can be confirmed on histopathology. In addition, a variety of histopathologies have been described depending on the cause and severity.

Authors response: To better reflect the way we utilized the histopathology we have changed the text to “Dogs were excluded if the histopathologic changes in the colonic biopsies were not consistent with AHDS [5, 8, 13]” (lines 110-111).

Line 210: It seems a little disingenuous to suggest that there was any alternative to using more than a single donor given the two study sites!

Authors response: The need to involve two institutions evolved because of a lower than expected incidence of AHDS that did not reasonably allow us to finish the project within the required funding period with one institution. Beyond that point, we think that using multiple donors reflects better real-life situations if FMT is to become more popular in the future.

Line 128: How did the authors define “healthy”. It is a nebulous concept, but at least the reader should know what boundaries the authors put on it.
Authors response: Healthy was defined as normal appetite, no vomiting, no diarrhea, no PU/PD, no cough, no chronic conditions or any other concerns noticed in the 4 weeks preceding sample collection. This is stated now in lines 140-142.

Line 133: Same comment as for line 104. More detail please. The authors should especially comment on the effect of the processing on the viability of oxygen-sensitive species.

Authors response: Stools were placed inside a Ziplock bag or a twist-top container and chilled on ice. Our protocol was designed to be consistent with how FMT would be used clinically by veterinarians. Our protocol is similar to human protocols for both fresh stool FMT and these protocols have been proven to be efficacious in patients^15,16^. In human FMT, it is acceptable to have up to 6 hours lag time from defecation to processing as long as the stool is kept in a closed container as we did. We agree that there will be some loss of strict anaerobes, especially from the processing of the feces prior to infusion. However, the increased presence of *Eubacterium*, *Faecalalibacterium* and *Prevotella* in FMT vs sham-treated dogs suggest that these anaerobes have survived in sufficient numbers to enable restoration of a more diverse microbiota.

Line 144: What pre/post-operative fasting/feeding protocol was used for the dogs?
What was the post-treatment diet? Surely that was standardized? At the very least, that needs to be reported, as any differences will be heavily influenced by colonic substrate, and perhaps all differences were due to dietary differences.

Authors response: Prior to colonoscopy, the dogs were fasted and only received medication according to the colon prep protocol. Post-procedure, they were fed a combination of boiled chicken and Hills I/D tinned diet (except for one control dog that was fed Royal Canin GI in hospital and at discharge; and one FMT recipient dog that was fed Royal Canin GI as she was fed Royal Canin adult pellets at home). All dogs ate at least once before discharge. We added a new paragraph (lines 167-171) indicating that.

Line 152: placed “immediately” into a cryovial? Was this after the processing (mixing, sieving, etc)? If it was not the same, then please discuss the potential for a) differences between bacteriome and viable bacteria, and b) differences between the bacteriome as described from this sample, and differences that can occur ex-vivo between defaecation and transplantation.

Authors response: We have addressed this above when the Reviewer raised similar concerns and made clarifications in the manuscript.

Line 158: Presumably the pathologist was blinded to group allocation. Please specify.

Authors response: Correct. This has been added to the manuscript (line 180).

Line 273: I’m not clear on what statistical test was used to examine the significance of the AHDS scores. It seems that there was a comparison between groups at each time point, and between timepoints within each group, but what about an overall model that accounts for baseline value? Friedman’s test?

Authors response: Nonparametric tests (Wilcoxon two sample test) were done in each group (within) to assess for differences between admission and discharge. Nonparametric tests (Wilcoxon two sample test) were also done between groups to assess for differences in clinical scores at admission and separately at discharge. We have adopted the Reviewer’s suggestion and also tested the Δ(discharge-admission) in clinical scores between groups (now reported in the results section lines 241-247).

Line 277: I would have wanted more clinical detail about these dogs. The AHDS scores are presented, but what was the range of clinical signs? What were their absolute Hct values, and did they differ between groups?

Authors response: We have added this information as requested (lines 303-312): “However, the difference in median (IQR) AHDS clinical score between discharge to admission of FMT treated dogs did not differ from that of sham-treated control dogs (-1.5 (3.5) vs. -5.5 (4.5), P=0.08; n=4). The mean (±SD) hematocrit of FMT recipient dogs did not differ from sham-treated control dogs on admission (65±1 vs. 67±12, P=0.85) and discharge (47±14 vs. 51±10, P=0.71). Similarly, the mean (±SD) total protein of FMT recipient dogs did not differ from sham-treated control dogs on admission (62±2 vs. 76±11, P=0.1) and discharge (52±7 vs. 49±13, P=0.76). The mean difference between admission and discharge did not differ between FMT recipient sham-treated control dogs for hematocrit (P=0.89) and total protein (P=0.31). Regardless of their group allocation, all the dogs with AHDS had a rapid and similar clinical recovery and left the hospital on the morning of the third day of hospitalization”

Line 280-281: Faecal grades?

Authors response: We graded the AHDS dog feces from 0-3 in accordance to the grading in the clinical score that was published by Morteir et al^14^.

Line 287: Was some sort of histopathological grade or score applied? If there was variation in severity and types of histological changes (e.g. regeneration, nature of infiltrate), then it would be interesting to see if they were associated with any particular bacteriome. I realise that this is a very small study, but it might be the basis of further hypothesis formulation.
The fact that some dogs had an eosinophilic infiltrate is demonstrative of the heterogeneity of this syndrome, and probably the variation in aetiology. This speaks against it being a homogenous dysbiosis that would be expected to be treated successfully with an essentially random FMT microflora.

Authors response: Table 2 represents the grading system we adapted from Unterer et al^10^ and applied in the present study. There was minimal variation within and between the groups.

The authors did not and do not claim that AHDS is associated with a homogenous dysbiosis. Previous literature and our study show that there are differences in alpha and beta diversity between AHDS dogs and healthy dogs.

Line 375: Given that this is not a defined disease, with a standard dysbiosis, and that clearly there was heterogeneity of disease in these dogs, focusing on the common differences ignores the possibility that there are pathogenic species / combinations in individuals. There is little the authors can do to analyse the data to avoid that, but perhaps there should be more discussion of that possibility.

Authors response: We agree with the Reviewer that it is possible that other pathogenic bacteria or combinations of pathogenic bacteria could in theory cause a similar clinical presentation (e.g., *Salmonella*, some *E. coli* strains) especially in a dynamic environment that is undergoing changes in intestinal microbial communities’ composition. That being said, we did not have evidence that this was the case in any of the dogs enrolled in this study and we prefer not to discuss it without evidence to support it.

Table 3. The authors really do need to note that they are trying to force a holistic understanding on a heterogeneous collection of starting bacteriomes, different aetiologies, and hence different disturbances. The authors cite the study by Suchodolski et al, but they fail to mention that not one of the “significantly different taxa” that defined AHDS in that study (e.g. Sutterella sp, C.perfringens, Ruminococcae, Blautia, etc), were significantly different in this present study, and vice versa! That is the key point to emphasise: the authors appear to be attempting to create a bacteriological definition of the disease and hence suggest a common treatment, yet that is, from the authors’ own data, demonstrably not the case.

Authors response: We acknowledge this point the discussion in lines 498-504: “However, in our study the same taxa did not differ between AHDS dogs and the healthy donors, whereas other SCFA-producing bacteria that were in low abundances in the present study were not described in the previous study. These differences between the studies emphasize the heterogeneity in the composition of gut microbial communities among dogs with AHDS which differ from each other with respect to their genetic background, immunological status, nutrition, and environmental factors, all of which are likely to be important factors that participate in the predisposition to this syndrome.”

Line 391-397: I don’t think it’s appropriate to make claims about the benefits of changes in a few genera of bacteria in relation to their metabolic products. The impact on the host is dependent on the entire microflora, and the substrate provided through the diet. There are overall quantitative and qualitative effects that completely supersede these relative changes. One bacteria may utilize one substrate, which may then be utilized to produce another substrate by another bacteria. The authors may be able to conjecture about SCFA production if they looked at the entire population in each dog, considering both the species and their absolute abundances, and then considered the population outputs given a common dietary substrate, but we shouldn’t make weak speculations based on a few relative changes.

Authors response: We appreciate the Reviewer’s comments, yet we think that the text as it is written does not make any claims. We merely described the type of bacteria that we found to have low abundances and that mostly belonged to a group of bacteria that produce short chain fatty acids which are considered important to gut health.

However, in the fourth discussion paragraph we also acknowledged the Reviewer’s point-of-view (lines 532-535): “However, changes in the content of gut SCFA cannot be predicted by changes in abundances of SCFA-producing bacteria by itself, and necessitates quantitative and qualitative analyses of fecal SCFA, and for the substrates required for SCFA production.”

Lastly, in the limitation paragraph (lines 561-563) we acknowledge it again and stated that we did not measure fecal concentrations of SCFAs, and thus we were unable to determine whether the increase in SCFA-producing bacteria was coupled with an increase in SCFA production “Fourthly, as we did not measure fecal concentrations of SCFAs, thus we were unable to determine whether the increase in SCFA-producing bacteria was coupled with an increase in SCFA production.”

Line 435: “referral hospital population”.

Authors response: We made the correction.

Line 442: It does not follow that simply because the histological scores were low, that the dogs were already recovering. And it was always going to be the case that it would be hard to demonstrate a clinical benefit, that authors surely knew that at the onset.

Authors response: We chose to work with AHDS because we thought that FMT would be most effective against a disease in which a central part of its pathogenesis (as the authors hypothesize) relates to a toxin-mediated intestinal injury. However, as the Reviewer noted, this is a clinical syndrome with a rapid clinical course which made it somewhat unfortunate with regards to our ability to see any clinical benefit **over a short term** and also the study was not designed to evaluate long term clinical outcomes (beyond 30 days).

In our opinion, the fact that all dogs had evidence of crypt regeneration means that they were already on an upslope to recovery. With that respect, we assume that the Reviewer would agree that the onset of clinical signs in most diseases is lagging behind the actual histological disease at the level of the affected tissues.

Line 446-447: Please avoid suggesting this is a single common disease. You are conflating what may be many different aetiologies.

Authors response: The authors are not trying to purport that AHDS has a single and unifying etiology, and do not think that this is reflected in our manuscript. We provided 6 citations that indicate an association between AHDS and *C. perfringens* overgrowth/toxins, and whilst this may differ from the Reviewer’s understanding of AHDS we do not think it is inappropriate to include these citations and their conclusions. We have added a sentence to highlight the syndromic nature of the disease (lines 485-486): “This highlights the syndromic nature of AHDS in which dogs share common clinical signs and clinicopathologic abnormalities but not necessarily a common etiology.”

Line 449-450: It is not appropriate to discuss this as if they are the same, as these dogs are different.

Authors response: We rephrased this part of the paragraph as follow (lines 487-490): “The histopathological changes in the intestine of dogs with AHDS resemble the histopathological lesions in the intestine of people with RCI [13, 49] and could support a pathomechanistic hypothesis of toxin-mediated mucosal injury in AHDS. **Aside from this similarity, the two diseases differ with respects to etiology, chronicity, and known predispositions**.”

We do not think that there is any problem with making a comparative contrast in this regard. We will defer to the editor decision on this point.

Line 453: AHDS is not “a defined thing”, that can have an effect on the microflora. And surely the authors are arguing in reverse – that AHDS is caused by a dysbiosis?

Authors response: Thank you for catching this. We changed the sentence (lines 495-496) to “Little is known about the fecal microbial composition and diversity in dogs with AHDS”

Line 454: Here the authors again are paltering with the concept that AHDS is a single entity. They cite the papers by Suchodulski and Markel, supposedly in support of a common dysbiosis, but fail to point out the more startling differences.

Authors response: We have changed the tense of the verb from ‘have’ to ‘had’ and started the sentence with (line 496): “In a previous study…” We hope that now it is clear that we do not support a single generalizable homogenous etiology/pathway that leads to AHDS. We also followed that sentence with the following (498-504): “However, in our study the same taxa did not differ between AHDS dogs and the healthy donors, whereas other SCFA-producing bacteria that were in low abundances in the present study were not described in the previous study. These differences between the studies emphasize the heterogeneity in the composition of gut microbial communities among dogs with AHDS which differ from each other with respect to their genetic background, immunological status, nutrition, and environmental factors, all of which are likely to be important factors that participate in the predisposition to this syndrome.”

Line 463: Are these simply epiphenomena, given the absence of any clinical difference or benefit?

Authors response: This study was designed to find a short-term clinical difference between two groups of dogs following a clinical intervention. The justification of sample size as we have shown, was based on a 48-hour difference in discharge from the hospital. Looking back at the only 2 large retrospective studies that exist, we think that the choice that we made is justifiable. However, knowing what we know today after this **pilot study**, we understand that a larger sample size is required to detect a difference (should one exist) that would be measured in a few hours rather than 2 days. **So, this was a long answer to say that neither we or the Reviewer could really know if this is an epiphenomenon or if the changes in microbial communities could actually have a significant clinical impact**.

Line 474-475: You cannot infer that as a conclusion: any effect of the FMT is entirely dependant on the microflora present in the donor. The benefits of an FMT may in the future be proven to be universal, as long as faecal quality is normal in the donor, but at the moment that seems very unlikely, and the examples of dysbiosis in otherwise apparently healthy dogs with normal faeces are legion. Thus, we will need, in the future, to define an appropriate donor microflora on the basis of the microflora, and not simply on the basis of the clinical appearance of normality.

Authors response: We accept the Reviewer’s comment and have toned down the message as follow (lines 521-523): “Hence, we **cautiously** suggest that a single FMT procedure in dogs with AHDS **might have the potential** to restore specific bacterial communities that are known to be important for intestinal health up to 30 days post-FMT (Figs. 6-8, Tables 4-5) [46, 53, 54].”

Lines 478-490: Again, the SCFA faecal concentrations cannot be predicted from looking at differences in a few genera. If the authors want to discuss this, and they are right to consider it, then they need to consider each individual dog’s entire microflora quantitatively and qualitatively, and then consider that in light of the diet being fed, and only then speculate as to what the faecal SFCA, let alone colonic luminal concentrations might be.

Authors response: We have added the following to reflect the Reviewer’s comment (lines 532-535): “However, changes in the content of gut SCFA cannot be predicted by changes in abundances of SCFA-producing bacteria by itself, and necessitates quantitative and qualitative analyses of fecal SCFA, and for the substrates required for SCFA production.”

However, as described by Zierer et al^17^, the fecal metabolome and fecal microbiome are intimately connected and profiling of the former explains most (on average 67.7±18.8%) of the variability in the latter.

Conclusions
I would have expected the authors to start with the conclusion that, in this small study, FMT had no clinical benefit. It should also include the statement that the significance of the bacteriome changes noted are of no known significance, given that this is not analogous to recurrent C.diff colitis in humans.

Authors response: The conclusion paragraph has been revised as follow (lines 569-573): “In this small pilot study FMT had no clinical benefit, however, a single FMT procedure resulted in a significant change in the fecal microbiome of FMT recipient dogs up to 1-month post-FMT. This change was characterized by increased abundance of SCFA-producing bacteria that, according to the literature, are beneficial for the overall intestinal health. Nonetheless, the clinical significance of the bacteriome changes noted in the present study remains to be determined.”

Figures – I hope that text will be easily readable in all figures. I did not find Figure 7 useful.
Authors response: The authors prefer to keep this figure as it clearly demonstrates significant changes in selected taxa that are considered by many references in the literature as taxa that are important to the overall health of the host.

**Concluding remarks by the authors to the Reviewer**: We thank the Reviewer for their meticulous review of the manuscript and many helpful suggestions. It is clear that the point-of-view of the Reviewer is different in some respects from that of the authors. Despite that, we did our best to adequately bring in and reflect the criticism of the Reviewer so that both faces of the coin will be adequately presented to the reader. We hope that by doing so we accommodated the major concerns that the Reviewer had.

**SECOND REVIEWER COMMENTS:**

Reviewer #1: In this manuscript entitled “One dog’s waste is another dog’s wealth: a pilot study of fecal microbiota transplantation in dogs with acute hemorrhagic diarrhea syndrome” the authors aimed to determine the effect of FMT on composition of fecal microbiota in dogs with AHDS. This study consists of a small number of AHDS dogs treated with FMT (n=4) and a control group (n=4). The manuscript is well written and the statistical analysis is robust. In its current state, this paper has limited clinical utility since no clinical improvement was observed with FMT treatment, however it provides valuable information about the microbial composition of dogs suffering from AHDS.

Major revisions:
1. There is a known association of AHDS and C. perfringens infection (<https://www.ncbi.nlm.nih.gov/pmc/articles/PMC6505910/>). This manuscript obtained histopathology but did not perform testing for C. perfringens and/or the presence of netF. To strength this paper, it is recommended that this information be added for the AHDS dogs. This helps to ensure the classification as AHDS.

Authors response:

In the study referenced by the Reviewer there were quite a few AHDS dogs that had a negative IHC for *Clostridium spp.* antigen (4/9 in duodenum and 4/9 in ileum, but 9/9 in the colon which is not really surprising given that it harbors many different clostridia), and only 5/9 had a positive PCR for NetF (whereas in the others *C. perfringens* was not isolated). Hence if the Reviewer is willing to accept the possibility (based on the referenced study) that some dogs with AHDS might not have a positive IHC but would still be considered to have the diagnosis of AHDS due to the clinical and clinicopathologic picture (as it was in the referenced study) then we do not see the value of doing IHC for *Clostridium* spp. We also contend that the presence of *C. perfringens* or NetF would merely suggest an association and not a cause-and-effect.

With much respect to both Reviewers, **one of their two major critical criticisms** of the other Reviewer was that **AHDS is not caused by a single etiology.** The point that we try to bring across is that two respected, knowledgeable expert Reviewers reviewed our manuscript and had very different opposing views on this very point. Hence, we respectfully ask the Reviewer to reconsider and withdraw the request for IHC for *Clostridium* spp. as we will not be able to accommodate it.

2. In the 16S data, there is no mention of C. perfringens, it is surprising that none of the 8 dogs with AHDS would not have had this organism present in the analysis? Was this OTU identified and just not mentioned? It is recommended that this be added to the manuscript as well.

Authors response: We added the following in lines 482-486: “In the present study, we did not find an increase in the abundance of *C. perfringens* in any of the AHDS dogs. While this is in contrast to several previous studies [5, 6, 9, 11, 13, 48] it is also consistent with other studies in which *C. perfringens* or its toxins were not increased [2, 4, 47]. This highlights the syndromic nature of AHDS in which dogs share common clinical signs and clinicopathologic abnormalities but not necessarily a common etiology.”

We also added a supplementary CSV file with all the taxa that were considered in the analyses and whether they were found significant or not. This way we hope that we are completely transparent with our results.

3. More information is needed for the FMT donor dogs. Was fecal infectious disease testing performed on donor dogs (such as the IDEXX Real PCR diarrhea panel)? Infectious disease testing in dogs should be standard of care and this is unclear in this manuscript.

Authors response: Donors were not screened by the IDEXX Real PCR. All donors in this study were healthy donors used by the respective universities. As mentioned in the donor paragraph (lines 140-145) they were vaccinated and routinely dewormed and had no clinical signs of intestinal dysfunction. Immediately before the fecal donation we screened them for CPV and worms with a fecal float. We think that in the context of the circumstances by which the dogs were maintained, and the preventative-medical measures taken, their use as FMT donors is justified ethically.

The authors also argue that submitting a sample from an FMT donor to IDEXX was impractical because by the time the result would have reached us the AHDS dog would have gone home. Also, to play the devil’s advocate, if this screening panel was done periodically, it does not provide any assurance that they would not have been reinfected with the pathogens screened by this panel (*Giardia* spp., *Cryptosporidium* spp., *Salmonella* spp., *Clostridium perfringens* enterotoxin A gene, canine enteric coronavirus, canine parvovirus 2 and canine distemper virus) just prior to stool donation.

4. Preparation for colonoscopy can have an effect a profound effect on the GIT (<https://www.nature.com/articles/s41598-019-40182-9>). Since the aim of the paper was to characterize the fecal microbiota of AHDS during disease and recovery it would have been helpful to include a group of AHDS that did not receive colonoscopy but underwent the same in hospital treatments.

Authors response: We acknowledge this as a limitation and discussed it in the discussion lines (565-567) as follow: “Lastly, our study design did not include a control group of AHDS dogs that did not undergo any treatment to ascertain what level of changes in microbial diversity was related to the colon cleansing procedure [70]”

However, it is of note that while in the Reviewer’s referenced study the changes in community composition was **restricted** **only to the immediate period of time after the colon prep**, in our study the altered bacterial community composition in AHDS dogs remained through admission, discharge, and 30 days after discharge, and are thus less likely to reflect an effect of the colonoscopy.

5. In-hospital treatment algorithms are not mentioned. This study would be strengthened by controlling what other treatments dogs received in the hospital. Additional information regarding treatments doing hospital stay and discharge is needed. Dietary history from hospital stay and discharge is also needed. Did the diet remain consistent throughout the study period for all AHDS dogs and donors?

Authors response: We addressed that when responding to the other Reviewer who had a similar comment. The treatment protocol was identical and included IV fluid to support hydration and adequate cardiovascular function.

Preoperatively the dogs were fasted and only received medication according to the colon prep protocol. Post-procedure they were fed a combination of boiled chicken and Hills I/D tinned diet (except for one control dog that was fed Royal Canin GI in hospital and at discharge; and one FMT recipient dog that was fed Royal Canin GI as she was fed Royal Canin adult pellets at home). All dogs ate at least once before discharge. We added a new paragraph (lines 1678-171) indicating that.

6. Added discussion needed about using 4 different donor dogs for this study and the variability that is seen overtime in donor dogs.

Authors response: That has been discussed in the following places in the manuscript:

Lines 131-133: “The nature of the study that involved 2 institutions necessitated utilizing fresh stool from different donors. The authors also think that using stool from multiple donors better simulates the variability in the composition of bacterial communities among the donors’ stool in a real-life FMT scenario.”

Lines 559-561: “Also, our results show that donor dogs’ microbiota clustered closely together indicating that there was only low variability in their microbiome diversity”

7. The authors compare AHDS to recurrent C. difficile infection (rCDI) in people. I do not feel this is a good comparison, since AHDS in most dogs is self-limiting and rCDI is not. It is recommended that this be de-emphasized in both the introduction and discussion as these diseases are not as similar as the authors described.

Authors response: This was a concern that the other Reviewer expressed, and we have de-emphasized and restated our references to *C. difficile* which is included in the substantial revision and restructuring of the introduction. We are confident that those changes throughout the manuscript will satisfy both Reviewers.

8. One dog with AHDS was on a diet that clinically is used in dogs with diet responsive chronic enteropathy (z/d), can the authors please provide additional information about this dog’s GI disease/history?

Authors response: This is another concern that was addressed in our response to the other Reviewer. We did not have any control over decisions that vets and owners made regarding the nutrition of the dogs in this study. Specifically, the dog on z/d diet had a history of skin allergies and this is why its primary care veterinarian decided to put it on z/d diet (i.e., food allergy). Over conditioning of one of the donor dogs (BCS 6/9) was the reason for putting it on the Hill’s metabolic diet.

9. Was fecal scoring (Purina and/or Waltham) completed? This should be included for both the donors and AHDS dogs at admission, discharge, and 30 days post.

Authors response: Consistent with a previous study from which we adapted our scoring system^14^, we scored feces from 0-3. On admission, all AHDS dogs had watery bloody diarrhea. All AHDS patients on 30-day post-discharge follow-up had firm stools equivalent to grade 3 on the Purina scale. The donors had a Purina scale grade 3 feces at all time points.

10. Figure 3: Changing these to traditional box and whisker plots (with individual data points within the box) would be helpful.

Authors response: We created a new figure with box and whisker plots (Figure 3).

11. Figure 6B. This heatmap is confusing. It may be helpful to perform unsupervised clustering on dogs instead of the OTUs, this would allow the reader to visualize that AHDS dogs were most similar to their donor.

Authors response: We have added a revised version of Fig 6B (according to the Reviewer’s recommendation) as a supplemental figure (S2 Fig).

12. In the discussion, it is recommended to include more information on the beneficial effects of restoring the microbiota (such as potential to prevent chronic enteropathy, etc.) since a clinical benefit was not observed in this small pilot.

Authors response: This is a controversial issue that we would prefer not to address in this manuscript. There is very little evidence that FMT (let alone a single FMT) provides any clinical benefit in people with Crohn’s Disease and Ulcerative Colitis– well defined clinical entities in which there were enough studies done to be interpreted by metanalyses that did not show a significant effect. In dogs it is also obfuscated by the mere fact that we do not have well defined definition of the ‘disease’. We do not think that it will contribute to this manuscript beyond what we already mentioned in the 4^th^ paragraph of the discussion (lines 525-535).

13. In the conclusions, be specific that no immediate clinical benefit (such as faster resolution of diarrhea, faster recovery, shorter hospitalization, etc.) was observed.

Authors response: The conclusion paragraph has been revised accordingly (lines 569-573).

Minor Revisions:
1. Line 50: Clostridium difficile is the incorrect and outdated name, this needs to be corrected (<https://pubmed.ncbi.nlm.nih.gov/27370902/>)

Authors response: correction was made.

2. Line 399: Correct “OUT” to “OTU”

Authors response: correction was made.

3. Line 403: Be specific about which potential pathobionts were observed

Authors response: Line 403 was an unfortunate editing error. We observed a significant increase in abundance of *Enterococcus* and *Shigella* at the genus level which were transiently increased in the sham-treated control dogs relative to FMT recipient dogs only at discharge. You can also find it in Fig. 6A and 6B. This information will also be available in the new supplementary CSV file that has all our results (S3 Table).

4. Line 447: Should be specific and list C. perfringens here.

Authors response: the change was made.

5. Lines 483-485: Be specific about which species these changes were documented in.

Authors response: the specific bacteria were added to the sentence.

6. S1 Table: Appetite and defecation are misspelled

Authors response: Corrections were made. Thank you!

REFERENCES USED FOR THIS REBUTTAL LETTER

1.	Burrows CF. Canine hemorrhagic gastroenteritis. J Am Anim Hosp Assoc 1977;13:451-458.

2.	Busch K, Suchodolski JS, Kuhner KA, Minamoto Y, Steiner JM, Mueller RS, Hartmann K, Unterer S. Clostridium perfringens enterotoxin and Clostridium difficile toxin A/B do not play a role in acute haemorrhagic diarrhoea syndrome in dogs. Vet Rec 2015;176:253.

3.	Cave NJ, Marks SL, Kass PH, Melli AC, Brophy MA. Evaluation of a routine diagnostic fecal panel for dogs with diarrhea. J Am Vet Med Assoc 2002;221:52-59.

4.	Heilmann RM, Guard MM, Steiner JM, Suchodolski JS, Unterer S. Fecal markers of inflammation, protein loss, and microbial changes in dogs with the acute hemorrhagic diarrhea syndrome (AHDS). J Vet Emerg Crit Care (San Antonio) 2017;27:586-589.

5.	Leipig-Rudolph M, Busch K, Prescott JF, Mehdizadeh Gohari I, Leutenegger CM, Hermanns W, Wolf G, Hartmann K, Verspohl J, Unterer S. Intestinal lesions in dogs with acute hemorrhagic diarrhea syndrome associated with netF-positive Clostridium perfringens type A. J Vet Diagn Invest 2018;30:495-503.

6.	Mehdizadeh Gohari I, Parreira VR, Nowell VJ, Nicholson VM, Oliphant K, Prescott JF. A novel pore-forming toxin in type A Clostridium perfringens is associated with both fatal canine hemorrhagic gastroenteritis and fatal foal necrotizing enterocolitis. PLoS One 2015;10:e0122684.

7.	Schlegel BJ, Van Dreumel T, Slavic D, Prescott JF. Clostridium perfringens type A fatal acute hemorrhagic gastroenteritis in a dog. Can Vet J 2012;53:555-557.

8.	Sindern N, Suchodolski JS, Leutenegger CM, Mehdizadeh Gohari I, Prescott JF, Proksch AL, Mueller RS, Busch K, Unterer S. Prevalence of Clostridium perfringens netE and netF toxin genes in the feces of dogs with acute hemorrhagic diarrhea syndrome. J Vet Intern Med 2019;33:100-105.

9.	Ziese AL, Suchodolski JS, Hartmann K, Busch K, Anderson A, Sarwar F, Sindern N, Unterer S. Effect of probiotic treatment on the clinical course, intestinal microbiome, and toxigenic Clostridium perfringens in dogs with acute hemorrhagic diarrhea. PLoS One 2018;13:e0204691.

10.	Unterer S, Busch. K, Leipig M, Hermanns W, Wolf G, Straubinger RK, Mueller RS, Hartmann K. Endoscopically visualized lesions, histologic findings, and bacterial invasion in the gastrointestinal mucosa of dogs with acute hemorrhagic diarrhea syndrome. J Vet Internal Med 2014;28:52-58.

11.	Schumacher VL, Martel A, Pasmans F, Van Immerseel F, Posthaus H. Endothelial binding of beta toxin to small intestinal mucosal endothelial cells in early stages of experimentally induced Clostridium perfringens type C enteritis in pigs. Vet Pathol 2013;50:626-629.

12.	Diab SS, Kinde H, Moore J, Shahriar MF, Odani J, Anthenill L, Songer G, Uzal FA. Pathology of Clostridium perfringens type C enterotoxemia in horses. Vet Pathol 2012;49:255-263.

13.	Mortier F, Strohmeyer K, Hartmann K, Unterer S. Acute haemorrhagic diarrhoea syndrome in dogs: 108 cases. Veterinary Record 2015;176:627-+.

14.	Mortier F, Strohmeyer K, Hartmann K, Unterer S. Acute haemorrhagic diarrhoea syndrome in dogs: 108 cases. Vet Rec 2015;176:627.

15.	Terveer EM, van Beurden YH, Goorhuis A, Seegers J, Bauer MP, van Nood E, Dijkgraaf MGW, Mulder CJJ, Vandenbroucke-Grauls C, Verspaget HW, Keller JJ, Kuijper EJ. How to: Establish and run a stool bank. Clin Microbiol Infect 2017;23:924-930.

16.	Lee CH, Steiner T, Petrof EO, Smieja M, Roscoe D, Nematallah A, Weese JS, Collins S, Moayyedi P, Crowther M, Ropeleski MJ, Jayaratne P, Higgins D, Li Y, Rau NV, et al. Frozen vs Fresh Fecal Microbiota Transplantation and Clinical Resolution of Diarrhea in Patients With Recurrent Clostridium difficile Infection: A Randomized Clinical Trial. JAMA 2016;315:142-149.

17.	Zierer J, Jackson MA, Kastenmuller G, Mangino M, Long T, Telenti A, Mohney RP, Small KS, Bell JT, Steves CJ, Valdes AM, Spector TD, Menni C. The fecal metabolome as a functional readout of the gut microbiome. Nat Genet 2018;50:790-795.
